# Oral Breathing Effects on Malocclusions and Mandibular Posture: Complex Consequences on Dentofacial Development in Pediatric Orthodontics

**DOI:** 10.3390/children12010072

**Published:** 2025-01-08

**Authors:** Dana Feștilă, Cristina Dora Ciobotaru, Tudor Suciu, Cristian Doru Olteanu, Mircea Ghergie

**Affiliations:** Department of Orthodontics, Faculty of Dentistry, “Iuliu Hațieganu” University of Medicine and Pharmacy, 400347 Cluj-Napoca, Romania; dana.festila@umfcluj.ro (D.F.); mircea.ghergie@umfcluj.ro (M.G.)

**Keywords:** oral breathing, mouth breathing, craniofacial development, posture, malocclusion, orthodontics

## Abstract

**Background/Objectives**: Oral breathing is a common condition, particularly in children, and it is associated with significant changes in craniofacial development, dentomaxillary anomalies, and overall health. Despite extensive research, the role of oral breathing in the development of malocclusion remains controversial, with debates on whether it is a causative factor or a secondary adaptation to existing craniofacial issues. **Methods**: This narrative review synthesizes studies published in the last 15 years, focusing on the impact of oral breathing on dentofacial development and mandibular posture. A comprehensive search was conducted on four electronic databases (Embase, Medline, ProQUEST, Scopus) using keywords related to oral breathing, malocclusion, mandibular posture, and craniofacial development. Studies were included if they focused on the effects of oral breathing on craniofacial morphology, malocclusion, and postural changes in children and adolescents aged 6–18 years. **Results**: Results indicate a strong link between oral breathing and dentofacial changes such as adenoid facies, Class II malocclusion, posterior crossbite, and anterior open bite. It causes cranial posture changes, particularly increased craniocervical extension, as a compensatory mechanism to maintain airway patency. **Conclusions**: Oral breathing is a risk factor for malocclusion prognosis, especially in growing children. Dentofacial changes in oral breathers include adenoid facies, convex facial profile, and increased lower facial height. Oral breathing also leads to significant changes in cranial posture, often accompanied by mandibular, lingual, and palatal alterations.

## 1. Introduction

Oral breathing, as a condition that affects individuals of all ages, particularly children [1,2], is not merely a behavioral habit [3,4,5]; it is a complex, multifaceted issue that intertwines with numerous aspects of craniofacial development and functional health [6]. Despite the significant research dedicated to understanding the mechanisms and consequences of oral breathing, there remains a degree of controversy surrounding its role in the development of malocclusion and its effect on the morphology and function of the maxillofacial region.

Oral breathing often results from upper airway obstruction, typically caused by factors such as adenoid hypertrophy, tonsillar enlargement, or anatomical abnormalities like narrowed nasal passages [1,7,8]. This disruption of normal respiratory function [9] compels the individual to shift from nasal breathing to oral breathing, which may be exacerbated by environmental factors [6,10], such as allergies, or by genetic craniofacial anomalies that predispose the individual to airway restriction. In these instances, oral breathing becomes not only a functional adaptation but also a contributing factor to the progressive development of malocclusion [3,8,9]. Consequently, it is necessary to consider both the etiological factors of oral breathing and its potential as a causative factor [7,11,12] in dentomaxillary anomalies, particularly when dealing with children whose craniofacial structures are still developing. Another aspect that complicates the relationship between oral breathing and malocclusion is the role of functional adaptation [1,4,13]. When nasal breathing is compromised, oral breathing serves as a compensatory mechanism, and its long-term presence can lead to changes in tongue posture, muscle function, and cranial posture [2,8,13,14]. This, in turn, affects the development of the maxilla, mandible, and teeth, contributing to skeletal and dental malocclusions [15]. Chronic oral breathing is often associated with a range of characteristic orofacial changes, including adenoid facies, lip incompetence, maxillary underdevelopment, and posterior crossbites [2,3,10]. It also affects cranial posture, with many oral breathers exhibiting increased craniocervical extension to maintain an open airway [5,16,17]. These changes can lead to the development of Class II malocclusions among other dental anomalies [8,18,19].

Considering the increasing evidence indicating that oral respiration can markedly affect craniofacial development, prompt diagnosis and intervention are essential [20]. Orthodontic treatment often becomes necessary when oral breathing leads to the development of malocclusion [20,21]. Identifying oral breathing early on provides the opportunity for intervention before skeletal changes become irreversible [3,22]. While orthodontic appliances have long been used to treat the dentomaxillary issues caused by oral breathing, the development of 3D printing technologies is opening new possibilities for individualized treatments. Traditional orthodontic devices, such as expanders and functional appliances [23,24,25,26] are effective in addressing many of the skeletal and dental issues associated with mouth breathing. However, the advent of 3D printing allows for the creation of customized, patient-specific devices [27,28,29,30] that can better address the unique anatomical needs of oral breathers. These new tools are an intriguing area of development in the field of pediatric orthodontics because they have the potential to significantly improve treatment outcomes and patient comfort.

This narrative review aims to consolidate the existing research concerning the effects of oral breathing on dentofacial development, mandibular position, and the consequent malocclusions by examining studies published over the last 15 years to clarify the complex relationship between oral breathing and its role in the development of these anomalies.

## 2. Materials and Methods

Eligibility Criteria: A narrative review of the literature was conducted to explore the correlation between oral breathing, dentomaxillary anomalies, and mandibular postural changes. In order to establish the eligibility criteria for the studies included in the review, the PICOS framework (population, intervention, comparison, outcome, and study design) was implemented (Table 1).

The exclusion criteria included articles in languages other than English, studies published before 2008, research conducted on adult population, and studies irrelevant to the correlation between oral breathing, dentomaxillary anomalies, and/or mandibular posture.

Information Sources and Search Strategy: Research, in order to identify relevant studies, was conducted on four electronic databases (Embase, Medline, ProQUEST, Scopus), as well as handsearching the grey literature. The search employed the following keywords: “oral breathing and mandibular posture changes”, “oral breathing and malocclusion”, “oral breathing and posture”, and “oral breathing and sagittal mandibular posture position”.

Selection and Data Collection Process: JabRef Bibliography Management v.5 [31] was used to save and automatically deduplicated references. Data were initially collected by a single author and the author committee cross-validated it to ensure accuracy. Academic debate helped resolve disagreements until a consensus was reached. Applying inclusion and exclusion criteria reduced the selection to 20 eligible articles. To increase transparency and organization in presenting our conclusions, the studies relevant to our research, the studies included in our review have been divided into two separate categories: studies examining the correlation between oral breathing and posture and studies analyzing dental and occlusal changes associated with oral breathing patterns.

Funding and Ethics Statement: This research was funded by “Iuliu Hațieganu” University of Medicine and Pharmacy, Cluj-Napoca. Given the nature of a literature review, no ethical approval was required.

## 3. Results

Although the initial database search retrieved 1578 articles, applying the inclusion and exclusion criteria narrowed the selection to a final set of 20 articles deemed eligible for inclusion. To improve clarity and structure in presenting our results, the studies relevant to our investigation of the impact of oral breathing on craniofacial development patterns, mandibular postural changes, and dentomaxillary anomalies will be divided into two categories: studies examining the correlation between oral breathing and posture, primarily mandibular posture changes (Table 2), and studies analyzing dental and occlusal changes associated with oral breathing patterns, specifically focusing on oral breathing and malocclusion (Table 3).

### 3.1. Correlation Between Oral Breathing and Posture

Cuccia et al. [16] conducted a study aimed at determining whether a relationship exists between oral breathing and head posture variables in children before these variables significantly influence craniofacial development. Abnormal head posture was noted to alter the load on multiple joints in the craniovertebral region, potentially leading to unfavorable dentofacial and craniofacial growth patterns.

The study included a sample of 35 children with oral breathing and 35 children with physiological (nasal) breathing, all of whom were consecutively admitted to the Department of Orthodontics at the University of Palermo for orthodontic treatment. The oral-breathing group consisted of 14 boys and 21 girls, all of whom had a history of mouth breathing confirmed by parental reporting and medical history. Clinical examinations revealed characteristic features, including lip incompetence at rest, dental crowding in the upper arch, “adenoid facies”, and a reduced maxillary transverse dimension, often associated with unilateral or bilateral crossbite. Breathing pattern evaluation in this group predominantly showed diaphragmatic inhalation, underexpansion of the thorax, and reduced nostril mobility, indicating diminished upper airway patency. Oral breathing was confirmed through the presence of condensed water vapor on a mirror placed near the mouth, although the underlying cause of oral respiration was not established. The physiological breathing group comprised 16 boys and 19 girls, randomly selected from children presenting with various orthodontic issues but without any history or clinical signs of mouth breathing. Exclusion criteria for both groups included prior or ongoing orthodontic treatment, vestibular or balance disorders, visual or hearing impairments, swallowing issues, spinal abnormalities (e.g., torticollis, scoliosis, or kyphosis), and craniofacial anomalies. For each participant, a lateral cephalogram was performed in the natural head position and subsequently evaluated trough cephalometric analysis.

The study found that oral breathing alters the muscular forces exerted by the tongue, cheeks, and lips on the maxillary arch. Common intraoral findings among oral-breathing children included a narrow maxillary arch with a high palatal vault, posterior crossbite, Class II or III dental malocclusion, and anterior open bite. A significant outcome was that children with nasopharyngeal airway obstruction exhibited a well-defined postural pattern characterized by reduced cervical lordosis and increased atlanto-occipital joint extension to maintain the horizontal Frankfurt plane. Cephalometric analysis revealed that oral-breathing children exhibited skeletal features such as an increased ANB angle and intermaxillary divergence (ANS-PNS/Go-Me), indicative of a dolichofacial Class II pattern. Oral breathers also demonstrated greater head extension, reduced cervical lordosis, and increased skeletal divergence compared to their nasal-breathing counterparts. Additionally, the hyoid bone was located in a lower position in oral-breathing patients.

A second study, conducted by Chambi-Rocha et al. [32] evaluates cephalometric differences in craniofacial structures, including the shape and position of the maxilla, mandible, upper airway, and hyoid bone, as well as head posture, among nasal-breathing and oral-breathing children and adolescents with normal facial growth patterns, while also employing a measurable diagnostic tool for determining the breathing pattern and subsequently following a rigorous patient selection process.

Participants were recruited during routine clinic visits at the College of Integrated Child Dentistry, University of Seville. The inclusion criteria specified boys and girls of white ethnicity, aged 7–16 years, with normal growth patterns and no neurological or congenital conditions, genetic syndromes, craniofacial deformities, severe systemic diseases, respiratory allergies, obstructive sleep apnea syndrome, or asthma. Exclusion criteria also included a history of upper airway surgery, orthodontic or orthopedic treatments, prolonged pacifier use (more than six months), prolonged bottle feeding (more than two years), and habits such as lip or finger sucking or anterior tongue thrusting. Of the evaluated participants, 98 met the inclusion criteria. To address bias factors, participants were divided into two age groups: G1 (7–9 years) and G2 (10–16 years). This division served three purposes: to distinguish craniofacial developmental changes influenced by breathing patterns from those due to normal growth; to account for occlusal changes, particularly changes in vertical facial dimensions associated with the replacement of mixed dentition by permanent teeth and not last to consider adenoid size reduction, which begins between ages 7 and 10, leading to nasopharyngeal size differences. Children under 7 years were excluded due to physiologically larger adenoids in both nasal and oral breathers, which might obscure differences in the adenoid area.

The study found no statistically significant differences in head posture between nasal-breathing and oral-breathing patients in either age group. However, in G2 (adolescents), oral breathers exhibited a lower position of the hyoid bone relative to the mandibular plane. Further analysis demonstrated that even among individuals with normal facial growth patterns, significant cephalometric differences were observed between oral and nasal breathers. Oral-breathing children showed reduced anteroposterior airway size compared to their nasal-breathing counterparts. In adolescents, additional differences were identified, including reduced lower facial height, an elongated palate, and a lower hyoid bone position relative to the mandibular plane.

This study reveals that even within a population with normal facial growth, oral-breathing individuals exhibit distinct differences in airway dimensions and craniofacial structures compared to nasal breathers, with variations becoming more pronounced in adolescents.

In 2010, Silveira et al. [5] conducted a study to evaluate age-related postural changes and their association with respiratory function in children with oral breathing patterns. The study involved 20 children, with 9 girls and 11 boys in the nasal-breathing group (mean age 8.6 years) and 7 girls and 10 boys in the oral-breathing group (mean age 8 years). The findings revealed a forward projection of the head and shoulders in oral-breathing children, consistent with a forward shift in the center of gravity. This shift was observed in 70% of the mouth-breathing group, while 70% of the nasal-breathing group maintained a normal center of gravity. The forward head posture and increased neck lordosis in oral breathers were attributed to changes in the stomatognathic system, which heightened tension in the head and shoulder muscles, altering their position in anteroposterior or lateral planes. Spirometry assessments indicated significantly reduced pulmonary function values in oral breathers compared to nasal breathers, highlighting the impact of mouth breathing on respiratory efficiency.

The study concluded that oral breathing modifies the stomatognathic system, resulting in altered posture and reduced lung function. These findings highlight the inter-relation of respiratory patterns, musculoskeletal alignment, and functional outcomes in children who breathe orally.

Following research, conducted by Roggia et al. [17], aimed at evaluating posture and body balance in students with and without oral breathing patterns. Furthermore, it explored correlations between postural assessments and sensory system function (visual, vestibular, and somatosensory) in oral breathers.

The final sample comprised 109 participants aged 8 to 12 years, including 51 oral breathers and 58 physiological breathers, with both sexes represented in each group. A descriptive analysis of the data revealed that mouth-breathing children exhibited a more anteriorly positioned head compared to their nasal-breathing peers. As a result of this anterior head positioning, oral breathers demonstrated an increased craniocervical angle, reduced cervical lordosis, elevated head posture, and greater head extension relative to the cervical spine. Regarding overall body posture, the only statistically significant difference observed was in the mean knee angle in the left lateral view. The oral-breathing group showed a higher tendency toward knee hyperextension (49%) compared to the control group (34%). A detailed evaluation of the relationship between head position and sensory system function revealed that greater head advancement correlated with increased impairment in visual, somatosensory, and vestibular systems. The anterior head positioning in oral breathers altered the mandibular resting position, occlusal contacts, and alignment of the optic and bipupillary planes. The study concluded that mouth-breathing participants exhibit significant postural changes, most notably in the knee position in the lateral view, as well as compromised body balance compared to the nasal-breathing group. Furthermore, a strong correlation was found between head positioning and sensory system dysfunction in mouth-breathing school children.

In 2011, Bolzan et al. [33] performed a comparative study, which evaluated facial types and head posture of nasal-breathing and oral-breathing children, distinguishing between habitual and obstructive causes, while also investigating the relationship between morphological facial index and head angulation in the sagittal plane. The study involved 59 children aged 8–11 years and 10 months, classified into three groups: nasal breathers, mouth breathers of obstructive etiology, and habitual mouth breathers. An otorhinolaryngologic evaluation confirmed the children’s breathing mode and etiology of mouth breathing. The study also assessed facial type and head postures to satisfy research purposes. Results showed a correlation between facial morphology and breathing mode/mouth breathing etiology by evidencing a predominance of short face and hyperbrachyfacial characteristics in nasal breathers and a predominance of long face and hyperdolichofacial and dolichofacial characteristics in mouth breathers. However, the study found no association between morphological facial index and head posture, stating that all 3 groups presented forward head postures, without difference among nasal breathers, obstructive mouth breathers, and habitual mouth breathers.

### 3.2. Occlusal Changes and Dentomaxillary Anomalies Resulting from Oral Breathing Patterns

A 2024 study [34] conducted by Topsakal et al. investigated morphological differences in cranial and dentofacial structures between individuals with mouth-breathing and nasal-breathing patterns. The researchers included 120 individuals, 60 in the nasal-breathing group with a mean age of 14.7 years, and 60 with a mean age of 14.3 years in the mouth-breathing group. Three-dimensional stereophotogrammetry, lateral cephalometric radiographs, and intraoral examination were used to determine the differences. Significant variations between mouth and nose breathers were found in this cross-sectional study, affecting both soft tissue and skeletal structures. Increased facial height, an adenoid facial type, and a retrognathic mandible were all supported by these variations. The authors also advise that orthodontists use the characteristic features seen in mouth-breathing anomalies for early detection and consider referring their patients for medical treatment of mouth breathing.

Grippaudo et al. [9] investigated the association between oral breathing and malocclusion, as well as other pathological oral habits, by applying the Risk of Malocclusion Assessment Index (ROMA) to a sample of 3017 school children. The study found a strong association between mouth breathing and various types of malocclusions, including sagittal stage abnormalities, anterior and posterior crossbites, and open bites. The findings indicated that mouth breathing and other harmful oral habits significantly contribute to the etiopathogenesis of malocclusion by disrupting the physiological balance necessary for normal growth. The study consistently observed significant associations between oral breathing and all occlusal issues evaluated, highlighting its pervasive negative impact on craniofacial development.

Similarly, Festa et al. [7] conducted a study to evaluate the association between upper airway obstruction and occlusal variables in children with a mean age of 6.2 ± 2.5 years. The study aimed to explore the cause–effect relationship between airway obstruction, caused by adenoidal or tonsillar hypertrophy and nasal septal deviation, and malocclusions. After applying exclusion criteria, the study included 221 participants, each of whom underwent a thorough dental examination performed by two orthodontic specialists with over 10 years of experience. The evaluated occlusal variables included canine relationship, posterior crossbite, open bite, deep bite, and alterations in sagittal step (either reduced or increased).

The study confirmed a high prevalence of malocclusion in mouth-breathing children compared to their nasal-breathing counterparts. However, no significant association was found between adenoidal hypertrophy and occlusal variables. This finding suggests that adenoidal hypertrophy may not directly contribute to malocclusions in children around the average age of 6 years, with malocclusions potentially developing at later stages. Conversely, the study identified strong associations between tonsillar hypertrophy and specific orthodontic issues: mild tonsillar hypertrophy (grade 2) was associated with Class II malocclusion and an increased sagittal step and severe tonsillar hypertrophy (grade 4) exhibited a robust association with malocclusion and increased sagittal step, suggesting a potential etiological role in the development of these issues. The findings suggest that malocclusion appears to have a dual role in the context of oral breathing: in cases of mild upper airway obstruction, malocclusion may act as a determinant in the onset of oral breathing. Conversely, in cases of severe tonsillar hypertrophy, malocclusion likely emerges as a consequence of the obstruction.

In 2021, İnönü-Sakallı et al. [35] conducted a study to assess the impact of adenotonsillar hypertrophy (ATH) on oral health, focusing on dental and periodontal health issues as well as on malocclusion. The study compared findings among three groups of children: those with adenotonsillar hypertrophy and mouth breathing (n = 40; patient group), those with nasal breathing (n = 40; control group), and those who had undergone adenotonsillectomy at least one year prior (n = 40; surgically treated group).

The study revealed that ATH was associated with significant dentofacial changes. A V-shaped narrowing of the maxillary arch and the presence of adenoid facies were significantly more common in the patient group compared to the control group. An open mouth posture was observed in approximately 80% of the children in the ATH group. Additionally, the patient group exhibited higher rates of anterior and inferior tongue positioning, along with a retrognathic mandibular position, highlighting the functional and structural impacts of ATH. Prominent maxillary anterior teeth were identified in 38% of children with ATH, compared to only 8% in the control group. The study also observed a significantly higher prevalence of Class II Division 1 malocclusion in the patient group. Although posterior crossbite and frontal open bite were slightly more frequent in the ATH group, these differences were not statistically significant. Overall, the findings suggest that adenotonsillar hypertrophy contributes to a range of dentofacial changes and a retrognathic mandibular position, thereby reinforcing the role of upper airway obstruction in the etiology of dentomaxillary anomalies.

Earlier on, Rossi et al. [36] aimed to investigate the dental and skeletal variables associated with craniofacial developmental disorders in mouth breathers, assessing the likelihood that these variables are related to the condition. This retrospective observational case–control study included a total sample of 1596 patients divided into three age groups: 5–12 years (n = 523), 13–18 years (n = 443), and 19–57 years (n = 340). The descriptive analysis revealed that Class II malocclusion was the most prevalent among the total sample. When stratified by age group and breathing mode, a pattern emerged. In the 5–12 years group, Class II malocclusion, a short and retrognathic mandible was significantly associated with mouth breathing, identifying these as risk factors. Conversely, decreased lower anterior facial height and a brachycephalic facial pattern were linked to nasal breathing and considered protective against oral breathing. In the 13–18 years group, Class II malocclusion, short mandibular length, and increased lower anterior facial height remained associated with mouth breathing, suggesting that the condition exacerbates during adolescence. Interestingly, adults showed no significant association between mouth breathing and skeletal variables. Instead, associations were observed only with dental variables, indicating that mouth breathing may not have a direct cause–effect relationship with skeletal changes in adults. These results support the age-dependent nature of craniofacial adaptations to mouth breathing, with more pronounced effects during childhood and adolescence.

Harari et al. [37] conducted a retrospective study, which aimed to investigate the impact of mouth breathing during childhood on craniofacial and dentofacial development in malocclusion patients treated in an orthodontic clinic. Clinical variables and cephalometric parameters of 116 pediatric patients, including 55 with nasal obstruction symptoms and oral breathing and 61 children with nasal breathing patterns, were evaluated. Results showed that mouth breathers experienced more backward and downward rotation of the mandible, increased overjet, and narrowing of upper and lower arches. The prevalence of a posterior cross bite was more frequent in mouth breathers (49% compared to 26%), and an abnormal lip-to-tongue anterior oral seal was more frequent in mouth breathers (56% compared to 30%). The study concluded that nasal respiratory obstruction with mouth breathing during critical growth periods in children leads to a higher tendency for clockwise rotation of the growing mandible, disproportionately increasing anterior lower vertical face height and decreasing posterior facial height.

In 2014, a study conducted by Chung Leng Munoz and Orta [38] aimed to compare cephalometric values between nasal and oral breathing children and measure upper and lower airway space. It involved 118 pediatric patients aged 6–12 years old, with 53 being oral breathers and 65 being nose breathers, with cephalometric tracing being performed for each lateral chephalometric radiograph. The study produced statistically significant results showing that mouth breathing children had a more retruded mandible, greater inclination of the mandibular plane and occlusal plane, and a smaller nasopharyngeal air space compared to nose breathing children. Oral breathing children also had a higher frequency of having the hyoid bone in a more elevated position and a higher tendency towards having a class II malocclusion compared to nasal breathing children.

A 2019 study conducted by Diouf et al. [39] aimed to compare the measurements of dental arches according to the grade and obstructive character of adenoids in 86 children aged between 8 and 12 years old. The results showed that subjects with obstructive adenoids were more likely to have a posterior mandibular arch length less marked. Additionally, subjects with obstructive adenoids were more likely to present more severe overbite than those in the non-obstructive adenoid group, where the overbite was in the range of normality. Subjects with a large grade of airway obstruction were significantly more likely to show an open bite than those with a moderate or small grade. The arch depth was also significantly more pronounced in subjects with large grades compared to those with small grades. In terms of qualitative variables, no significant difference was found between the subjects who presented obstructive adenoids and those who did not. However, the study found that subjects with obstructive adenoids were more likely to have a posterior mandibular arch length that is less marked and a more severe overbite than those in the non-obstructive adenoid group.

The same year, Poddębniak et al. [40] investigated the incidence of open bite in children with chronic oral breathing due to adenoid hypertrophy. The study involved 236 pediatric patients aged 7–12 years, with an oral breathing group consisting of 93 children with adenoid hypertrophy. The control group consisted of 143 children without nasal breathing obstacles. The results showed that partial anterior open bite was more common in the oral breathing group (11.82%) patients, compared to the control group (4.2%) patients. A statistically significant difference was observed in the incidence of open bite between the oral breathing group and the control group. The study concluded by stating that habitual oral breathing due to adenoid hypertrophy is a major cause of open bite development.

In a cross-sectional study, Noor et al. [41] evaluated the orthodontic records of 62 patients (29 males and 33 females) with erupted first permanent molars. The study found a significantly higher proportion of female mouth breathers than males. Mouth breathing was more prevalent among patients with angle Class II and Class III malocclusions compared to nasal breathers, though this finding was not statistically significant. Class I malocclusion was more common among nasal breathers, with variations observed between the right and left sides of the oral cavity. A notable 87.5% of oral breathers in this study routinely relied on mouth breathing as their primary mode of respiration. While the association between mouth breathing and angle malocclusion was not statistically significant, other findings included open lip posture, a prominent lower lip with a functionally compromised upper lip, and gingival changes such as hypertrophy and bleeding. These observations indicate that mouth breathing affects orofacial structures, particularly lip function and gingival health.

Milanesi et al.’s 2018 study [42] explored variables associated with the diagnosis of mouth breathing across multiple disciplines, including speech–language pathology, otolaryngology, dentistry, and orthodontics. The study involved 119 children (64 boys and 55 girls), with 49 in the nasal-breathing group and 70 in the mouth-breathing group. The study identified several features strongly associated with mouth breathing. Children with a convex facial profile were 3.78 times more likely to exhibit mouth breathing compared to those with a straight profile, while an obtuse nasolabial angle increased the likelihood by 4.30 times. Similarly, an open lip posture and a tongue resting on the buccal floor were strongly predictive of mouth breathing, with odds ratios of 4.13 and 5.88, respectively. During mastication, the presence of excessive contraction of the mentalis and orbicularis oris muscles was observed, leading to incomplete lip closure and tongue interposition. This group also showed reduced hard palate width, obstructive pharyngeal tonsils, and increased prevalence of angle Class II malocclusion. The study highlights the multifactorial nature of mouth breathing, emphasizing its impact on stomatognathic function and craniofacial growth.

Souki et al. [43] conducted a 2009 epidemiologic study to report the prevalence of malocclusion among children and its association with upper airway obstruction due to adenoids, tonsillar hyperplasia, and allergic rhinitis. The study included 401 children, of whom 71.8% had adenoid or tonsillar obstruction, 18.7% had allergic rhinitis, and 9.5% exhibited non-obstructive mouth breathing. Posterior crossbite was observed in 30% of children with mixed dentition and increased to 48% in those with permanent dentition. Anterior open bite and Class II malocclusion were common in both mixed and permanent dentition stages. However, the study found no significant association between upper airway obstruction and malocclusion in terms of Class II malocclusion, anterior open bite, or posterior crossbite. Despite these findings, posterior crossbite and open bite were more prevalent in mouth breathers, though not all oral-breathing children fit the expected “oral-breathing dental stereotype”.

In 2014, Basheer et al. [44] examined dental and soft tissue anomalies in oral breathing children with and without adenoid hypertrophy. The study involved 50 children aged 6–12 years divided into three groups: 20 mouth breathers with hypertrophic adenoids and 60% nasopharyngeal obstruction, 20 mouth breathers without nasal obstruction, and 10 nasal breathers as controls. The study identified significant proclination of upper and lower incisors in both groups of mouth breathers. Children with hypertrophic adenoids displayed greater facial convexity, increased depth of the labial-mental fossa, and a larger interlabial gap compared to nasal breathers. These findings support the pre-existing data on the impact of nasopharyngeal obstruction on craniofacial development, particularly during the 6–12 years age range.

In 2023, Borsa et al. conducted a cross-sectional study [45] involving 359 11-year-old children to explore the relationship between oral parafunctions, such as low tongue position and oral breathing, and the development of malocclusions. The study revealed that mouth breathing and low tongue position were significant predictors of malocclusion. Open cleft lips during rest were frequently observed in mouth breathers, linked to mandibular protrusion, maxillary underdevelopment, and hyperdivergent facial patterns. The study emphasized the pathologic nature of mouth breathing, noting again its role in inducing craniofacial changes such as adenoid facies, underdeveloped maxilla, and open occlusion. It also reinforces the role of seasonal allergies as a potential contributing factor to the high prevalence of oral breathing in the study population.

Similarly, in 2015, Agostinho et al. [46] evaluated dental positions, skeletal effects, and pharyngeal airway space in children with chronic allergic rhinitis compared to a control group of nasal breathers. The study, conducted on 70 children aged 5–14 years, found that oral breathers with allergic rhinitis exhibited increased lower facial height, a steeper mandibular plane angle, and reduced airway dimensions, supporting the impact of allergic rhinitis on craniofacial development and malocclusion patterns.

## 4. Discussion

Over the years, research has consistently highlighted its potential role in malocclusion and changes to maxillofacial morphology [8,14,19,38], but the full extent of its effects is still being understood. The complexity of oral breathing lies not only in its impact on dental and skeletal structures but also in how it interacts with and exacerbates [8,15] other developmental and environmental factors. While the majority of studies confirm the associations mentioned in the existing literature, some offer differing viewpoints [32,41,43]. The complexities of this issue arise from the variety of factors that concur in oral breathing, which can be either a contributing factor to malocclusion or a consequence of pre-existing craniofacial structural anomalies [7,11,12,47]. In clinical examinations of patients with oral breathing, several characteristic features are consistently observed, including adenoid facies, lip incompetence at rest, upper arch dental crowding, and reduced maxillary transverse dimension, with associated unilateral or bilateral crossbite [16]. Other common signs include frontal open bite [40,43], an increased mentolabial sulcus depth [44], an open labial gap [35,42], maxillary anterior tooth protrusion, and a convex facial profile [42,44]. The convex facial type has been consistently associated with oral breathing, as well as alterations in masticatory patterns, such as unilateral chewing, excessive contraction of the mentalis and perioral muscles during mastication, and forward movement of the tongue and head during swallowing [42]. These findings strongly suggest that oral breathing may lead to specific functional adaptations in the craniofacial complex, with significant effects on both dental occlusion and facial morphology. When it comes to malocclusion, studies have shown a higher prevalence of occlusal abnormalities in children who breathe through the mouth [7]. Specifically, Class II malocclusion is commonly observed among oral breathers [16,38]. In this context, oral breathing and associated vicious habits are considered important risk factors for the development of malocclusion, as they disrupt the physiological balance of growth and craniofacial development [9]. Notably, abnormal breathing patterns (such as mixed or exclusively oral breathing) and low tongue posture at rest are found to increase the risk of developing malocclusion by up to three times [45].

While early detection and treatment of oral breathing were not explicit variables in this study, their importance is emphasized in the context of the reviewed literature by being essential for avoiding the long-term consequences of these developmental problems [34,40]. The earlier oral breathing is addressed, the better the chances of mitigating its effects [13,20,26,28] on the maxillofacial complex and, as such, this point is discussed as a remark derived from the reviewed literature rather than as a direct finding of this review. Orthodontic interventions are most effective when introduced during the growth period before skeletal changes become more rigid [21,35,44,48]. Early detection and treatment of oral breathing can reduce the need for invasive orthodontic procedures later on, especially in cases of severe malocclusion. Moreover, early intervention can help prevent the development of compensatory habits, such as tongue thrusting, mouth breathing during speech, and pathological mastication patterns [7,20,21,48], which may further complicate treatment outcomes. Comprehensive multidisciplinary care [42,49], involving orthodontists, pediatricians, ENT specialists, and speech therapists, is essential for managing oral breathing and its impact on dental and skeletal development [21,34,50,51].

Another important consideration is the need to distinguish between the primary causes of oral breathing and secondary effects in cases where oral breathing arises from genetically transmitted craniofacial anomalies—such as vertical maxillary excess (VME) or skeletal open bite [4,7,11,12]—and cases where oral breathing itself leads to the development of malocclusion. In the former scenario, children with vertical maxillary excess or similar skeletal conditions often have narrowed upper airways, making nasal breathing difficult. In these cases, the presence of oral breathing is primarily a compensatory mechanism rather than a causative factor. The upper airway obstruction due to the genetically shaped dentofacial complex results in the need for mouth breathing, which further exacerbates malocclusion over time. This nuanced difference [1,7] is critical when evaluating the cause and effect of oral breathing on malocclusion. The distinction between the primary causes of oral breathing and its secondary effects is essential, as this differentiation may shape future research and encourage the development of more tailored treatment strategies. This aspect was not specifically addressed in the current review; however, it is a significant issue that requires further investigation. For future research, it may be essential to design a study that differentiates these two pathways to malocclusion, as they may require distinct therapeutic approaches. Additionally, oral breathing is associated with significant changes in cranial posture in relation to the spine [5,32,33,52]. Specifically, oral breathing has been linked to an increased atlanto-occipital joint extension, which serves to maintain the Frankfurt horizontal plane [16]. This postural adaptation is seen in several studies, which have indicated that oral breathing causes increased craniocervical extension to enlarge the airway and enhance oropharyngeal patency, with corresponding changes in mandibular posture, lingual posture, and even the position of the soft palate [16].

One of the major morphological consequences of oral breathing is the potential underdevelopment of the maxilla in children [53]. This is linked to mandibular and maxillary rotation backward and downward, causing a steep occlusal plane and resulting in increased lower facial height, reduced maxillary and mandibular length [37,39], low overbite, and a narrower upper airway [45,46]. Oral breathers often display forward projection of the head and shoulders, which is associated with a forward shift in the center of gravity. This postural change, in turn, influences the head and shoulder muscles, increasing muscle tension and altering their position in anteroposterior and lateral directions [5]. Furthermore, studies have shown that the degree of head advancement in mouth-breathing children correlates with increased visual, somatosensory, and vestibular impairments. As the head is positioned forward due to oral breathing, mandibular resting position and occlusal contacts are altered, particularly in the optic and bipupillary planes [17]. These changes are indicative of the broader functional impact that oral breathing has on craniofacial development and occlusal relationships.

Limitations arise, since the inclusion of studies with varying methods, sample sizes, and criteria for diagnosing oral breathing poses a challenge to comparing results and forming consistent conclusions. Additionally, as observed, our research did not explore the potential role of genetic predispositions and environmental factors in the development of oral breathing and related anomalies, leaving an important aspect of the condition unaddressed. This suggests the importance of future research to address these knowledge gaps and improve our understanding of oral breathing and its impact in craniofacial development.

## 5. Conclusions

Oral breathing can be considered a significant risk factor for the prognosis of malocclusion. The dentofacial changes observed in oral breathers include several characteristic features: adenoid facies, a convex facial profile, increased lower facial height, flared nostrils, lip incompetence, dry lips, increased depth of the mentolabial sulcus, maxillary anterior tooth protrusion, increased sagittal step, upper dental arch crowding, retrognathism, frontal open bite, a narrow maxilla with protrusion, and distalized occlusion. Additionally, other issues such as unilateral or bilateral crossbites, marginal gingivitis, and cervical caries on the upper incisors are also common among oral breathers. Oral breathing can also lead to significant changes in cranial posture with increased craniocervical extension, which serves to enlarge the airway and improve oropharyngeal patency. These postural changes are frequently accompanied by mandibular, lingual, and soft palate alterations.

## Figures and Tables

**Table 1:** Eligibility criteria.

Domain	Inclusion Criteria
Population	Children aged 6–12 years and adolescents aged 13–18 years.
Intervention	Studies examining the impact of oral breathing on craniofacial development, specifically dentomaxillary anomalies and mandibular postural changes.
Comparison	Comparisons were drawn between oral breathing and nasal breathing populations or against normal craniofacial development patterns.
Outcome	The primary outcomes included correlations and clinical insights into the effects of oral breathing on dentomaxillary anomalies and mandibular posture, with a focus on orthodontic and postural implications
Study Design	Observational, cross-sectional, cohort, experimental prospective controlled studies (randomized and non-randomized), and case–control studies published in the last 15 years.

**Table 2:** Correlation between oral breathing and posture.

Study, Year	Population	Outcomes
Cuccia et al., 2008 [16]	Total n = 70 Oral breathers (n = 35) Nasal breathers (n = 35)	In oral breathers there is a well-defined postural pattern characterized by greater head extension, reduced cervical lordosis, and increased skeletal divergence compared to their nasal-breathing counterparts. Cephalometric analysis revealed that oral-breathing children exhibited skeletal features such as an increased ANB angle and intermaxillary divergence (ANS-PNS/Go-Me), indicative of a dolichofacial Class II pattern.
2. Chambi-Rocha et al., 2018 [32]	Total n = 98 Oral breathers (n = 56) Nasal breathers (n = 42)	Even within a population with normal facial growth, oral-breathing individuals exhibit distinct differences in airway dimensions and craniofacial structures compared to nasal breathers, with variations becoming more pronounced in adolescents (reduced lower facial height, an elongated palate, and a lower hyoid bone position relative to the mandibular plane).
3. Silveira et al., 2010 [5]	Total n = 37 Oral breathers (n = 20) Nasal breathers (n = 17)	The findings revealed a forward projection of the head and shoulders with an increased neck lordosis in oral-breathing children, consistent with a forward shift in the center of gravity.
4. Roggia et al., 2016 [17]	Total n = 109 Oral breathers (n = 51) Nasal breathers (n = 58)	Oral breathers presented an anterior head positioning and demonstrated an increased craniocervical angle, reduced cervical lordosis, elevated head posture, and greater head extension relative to the cervical spine.
5. Bolzan et al., 2011 [33]	Total n = 59 Oral breathers (n = 15) Nasal breathers (n = 44)	Oral breathers presented a longer face compared to nasal breathers, which were more likely to have brachyfacial features; head posture seemed to be unaffected by breathing type, mouth breathing etiology, or facial type.

**Table 3:** Dental and occlusal changes associated with oral breathing patterns.

Study, Year	Population	Outcomes
Topsakal et al., 2024 [34]	Total n = 120 Oral breathers (n = 60) Nasal breathers (n = 60)	Significant variations between mouth and nose breathers were found in this cross-sectional study, affecting both soft tissue and skeletal structures. Increased facial height, an adenoid facial type, and a retrognathic mandible were all supported by these variations.
2. Grippaudo et al., 2016 [9]	Total n = 3017 Oral breathers (n = na) Nasal breathers (n = na)	The study consistently observed significant associations between oral breathing and all occlusal issues evaluated (sagittal anomalies, anterior and posterior crossbites, open bite), highlighting its pervasive impact on craniofacial development.
3. Festa et al., 2021 [7]	Total n = 221 Oral breathers (n = na) Nasal breathers (n = na)	In cases of mild upper airway obstruction, malocclusion may act as a determinant in the onset of oral breathing. Conversely, in cases of severe airway obstruction, malocclusion likely emerges as a consequence.
4. İnönü-Sakalli et al., 2021 [35]	Total n = 120 Oral breathers (n = 40) Nasal breathers (n = 40) Ex Oral breathers (n = 40)	The findings suggest that adenotonsillar hypertrophy contributes to a range of dentofacial changes, including V-shaped maxillary arch narrowing, adenoid facies, macroglossia, an open labial cleft, anterior and inferior tongue positioning, and a retrognathic mandibular position, as well as a significantly higher prevalence of Class II Division 1 malocclusion in the oral breathing group.
5. Rossi et al., 2015 [36]	Total n = 1596 Oral breathers (n = na) Nasal breathers (n = na)	The descriptive analysis revealed that Class II malocclusion was the most prevalent among the total sample. Class II malocclusion, short mandibular length, and increased lower anterior facial height remained associated with mouth breathing, suggesting the age-dependent nature of craniofacial adaptations to mouth breathing, with more pronounced effects during childhood and adolescence.
6. Harari et al., 2010 [37]	Total n = 116 Oral breathers (n = 55) Nasal breathers (n = 61)	Nasal respiratory obstruction with mouth breathing during critical growth periods in children leads to a higher tendency for clockwise rotation of the growing mandible, disproportionately increasing anterior lower vertical face height and decreasing posterior facial height.
7. Chung Leng Munoz and Orta, 2014 [38]	Total n = 118 Oral breathers (n = 53) Nasal breathers (n = 65)	Oral breathing children had a more retruded mandible, greater inclination of the mandibular plane and occlusal plane, and a smaller nasopharyngeal air space compared to nose breathing children. Oral breathing children also had a higher frequency of having the hyoid bone in a more elevated position and a higher tendency towards having a class II malocclusion.
8. Diouf et al., 2019 [39]	Total n = 1596 Oral breathers (n = na) Nasal breathers (n = na)	Subjects with obstructive adenoids and oral breathing pattern were more likely to have a posterior mandibular arch length less marked and more severe overbite than those in the non-obstructive adenoid group
9. Poddębniak et al., 2019 [40]	Total n = 1596 Oral breathers (n = na) Nasal breathers (n = na)	A statistically significant difference was observed in the incidence of open bite between the oral breathing group and the control group.
10. Noor et al., 2021 [41]	Total n = 62 Oral breathers (n = na) Nasal breathers (n = na)	Mouth breathing was more prevalent among patients with angle Class II and Class III malocclusions compared to nasal breathers, though this finding was not statistically significant.
11. Milanesi et al., 2018 [42]	Total n = 119 Oral breathers (n = 49) Nasal breathers (n = 70)	The oral breathing group showed reduced hard palate width, obstructive pharyngeal tonsils, and increased prevalence of angle Class II malocclusion
12. Souki et al., 2009 [43]	Total n = 401 Oral breathers (n = 401) Nasal breathers (n = 0)	However, the study found no significant association between upper airway obstruction and malocclusion in terms of Class II malocclusion, anterior open bite, or posterior crossbite. Despite these findings, posterior crossbite and open bite were more prevalent in mouth breathers, though not all oral-breathing children fit the expected “oral-breathing dental stereotype”
13. Basheer et al., 2014 [44]	Total n = 50 Oral breathers—airway obstruction (n = 20) Oral breathers—no airway obstruction (n = 20) Nasal breathers (n = 10)	The study identified significant proclination of upper and lower incisors in both groups of mouth breathers. Children with hypertrophic adenoids displayed greater facial convexity, increased depth of the labial-mental fossa, and a larger interlabial gap compared to nasal breathers, implying the impact of nasopharyngeal obstruction on craniofacial development, particularly during the 6–12 years age range.
14. Borsa et al., 2023 [45]	Total n = 359 Oral breathers (n = na) Nasal breathers (n = na)	The study emphasized the pathologic nature of mouth breathing, noting its role in inducing craniofacial changes such as adenoid facies, underdeveloped maxilla, and openbite.
15. Agostinho et al., 2015 [46]	Total n = 70 Oral breathers (n = 35) Nasal breathers (n = 35)	Oral breathers with allergic rhinitis exhibited increased lower facial height, a steeper mandibular plane angle, and reduced airway dimensions, with oral breathing having an impact on craniofacial development and malocclusion patterns.

na = non applicable

## Data Availability

Data sharing not applicable. No new data were created or analyzed 659 in this study. Data sharing is not applicable to this article.
